# Dimethyl 1,4-dihydro-4-(4-methoxy­phen­yl)-2,6-dimethyl­pyridine-3,5-dicarboxyl­ate

**DOI:** 10.1107/S1600536810004940

**Published:** 2010-02-13

**Authors:** Wan-Sin Loh, Hoong-Kun Fun, B. Palakshi Reddy, V. Vijayakumar, S. Sarveswari

**Affiliations:** a X-ray Crystallography Unit, School of Physics, Universiti Sains Malaysia, 11800 USM, Penang, Malaysia; b Organic Chemistry Division, School of Advanced Sciences, VIT University, Vellore 632 014, India

## Abstract

In the title compound, C_18_H_21_NO_5_, the dihydro­pyridine ring adopts a flattened-boat conformation and its planar part forms a dihedral angle of 84.60 (2)° with the benzene ring. In the crystal, inter­molecular N—H⋯O and C—H⋯O hydrogen bonds result in the formation of zigzag layers parallel to (001). These layers are inter­connected *via* C—H⋯π inter­actions.

## Related literature

For the synthesis, see: Rathore *et al.* (2009 ▶). For general background and applications of 1,4-dihydro­pyridine derivatives, see: Bocker & Guengerich (1986 ▶); Cooper *et al.* (1992 ▶); Gaudio *et al.* (1994 ▶); Gordeev *et al.* (1996 ▶); Sunkel *et al.* (1992 ▶); Vo *et al.* (1995 ▶). For ring conformations, see: Cremer & Pople (1975 ▶). For bond-length data, see: Allen *et al.* (1987 ▶). For related structures, see: Fun *et al.* (2009*a* ▶,*b* ▶). For hydrogen-bond motifs, see: Bernstein *et al.* (1995 ▶). For the stability of the temperature controller used for the data collection, see: Cosier & Glazer (1986 ▶).
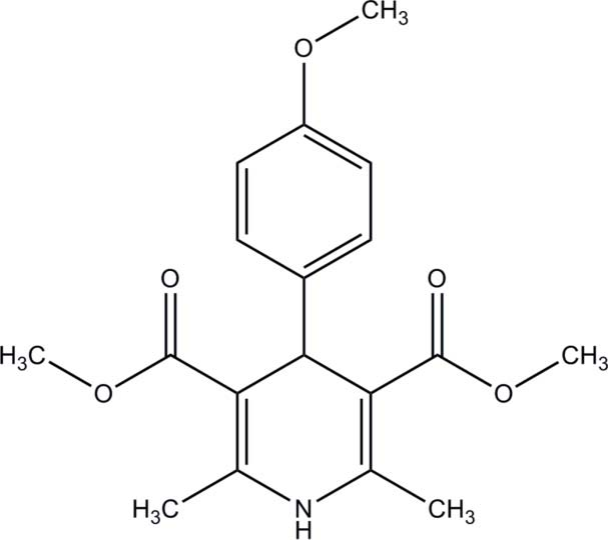


## Experimental

### 

#### Crystal data

C_18_H_21_NO_5_
*M* *_r_* = 331.36
Triclinic, 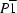

*a* = 7.4106 (3) Å
*b* = 9.5715 (5) Å
*c* = 11.7771 (6) Å
α = 83.029 (1)°
β = 83.834 (1)°
γ = 77.424 (1)°
*V* = 806.46 (7) Å^3^
*Z* = 2
Mo *K*α radiation
μ = 0.10 mm^−1^
*T* = 100 K
0.35 × 0.34 × 0.24 mm

#### Data collection

Bruker SMART APEX DUO CCD area-detector diffractometer
Absorption correction: multi-scan (*SADABS*; Bruker, 2009 ▶) *T*_min_ = 0.966, *T*_max_ = 0.976
19600 measured reflections
4664 independent reflections
4319 reflections with *I* > 2σ(*I*)
*R*_int_ = 0.019

#### Refinement

*R*[*F* ^2^ > 2σ(*F* ^2^)] = 0.037
*wR*(*F* ^2^) = 0.110
*S* = 1.05
4664 reflections
226 parameters
H atoms treated by a mixture of independent and constrained refinement
Δρ_max_ = 0.48 e Å^−3^
Δρ_min_ = −0.22 e Å^−3^

### 

Data collection: *APEX DUO* (Bruker, 2009 ▶); cell refinement: *SAINT* (Bruker, 2009 ▶); data reduction: *SAINT*; program(s) used to solve structure: *SHELXTL* (Sheldrick, 2008 ▶); program(s) used to refine structure: *SHELXTL*; molecular graphics: *SHELXTL*; software used to prepare material for publication: *SHELXTL* and *PLATON* (Spek, 2009 ▶).

## Supplementary Material

Crystal structure: contains datablocks global, I. DOI: 10.1107/S1600536810004940/ci5029sup1.cif

Structure factors: contains datablocks I. DOI: 10.1107/S1600536810004940/ci5029Isup2.hkl

Additional supplementary materials:  crystallographic information; 3D view; checkCIF report

## Figures and Tables

**Table 1:** Hydrogen-bond geometry (Å, °) *Cg*1 is the centroid of the C1–C6 ring.

*D*—H⋯*A*	*D*—H	H⋯*A*	*D*⋯*A*	*D*—H⋯*A*
N1—H1*N*1⋯O4^i^	0.87 (2)	2.23 (2)	3.0906 (10)	169 (1)
C14—H14*A*⋯O1^ii^	0.96	2.54	3.4631 (13)	162
C18—H18*B*⋯O4^i^	0.96	2.56	3.4558 (12)	156
C12—H12*B*⋯*Cg*1^iii^	0.96	2.79	3.6549 (11)	151

Symmetry codes: (i) 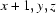; (ii) 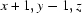; (iii) 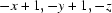.

**Fig. 1.**
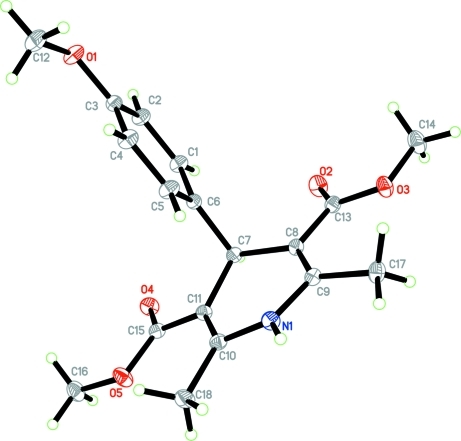
The molecular structure of the title compound, showing 50% probability displacement ellipsoids and the atom-numbering scheme.

**Fig. 2.**
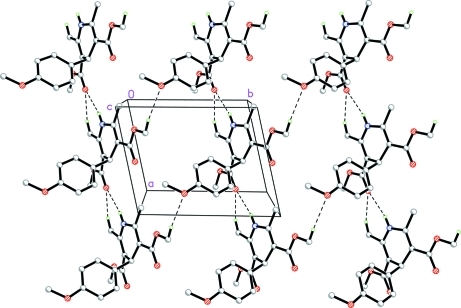
The crystal packing of the title compound, viewed approximately along the c axis, showing R12(6) ring motifs. H atoms not involved in hydrogen bonding (dashed lines) have been omitted for clarity.

## supplementary crystallographic information

### Comment

Hantzsch 1,4-dihydropyridines (1,4-DHPS) are biologically active compounds which include various vasodilator, antihypertensive, bronchodilator, heptaprotective, antitumor, antimutagenic, geroprotective and antidiabetic agents (Gaudio *et al.*, 1994). Nifedipine, nitrendipine and nimodipine have been used commercially as calcium channel blockers (Bocker & Guengerich, 1986; Gordeev *et al.*, 1996). For the treatment of congestive heart failure a number of DHP calcium antagonists have been introduced (Sunkel *et al.*, 1992; Vo *et al.*, 1995). Some DHPs have also been introduced as neuroprotectant and cognition enhancers. In addition, a number of DHPs with platelet anti-aggregatory activity have also been discovered (Cooper *et al.*, 1992).

In the title compound (Fig. 1), the 1,4-dihydropyridine ring (C7–C11/N1) adopts a flattened-boat conformation with puckering parameter Q = 0.2368 (9) Å; Θ = 72.8 (2)° and φ = 186.1 (2)° (Cremer & Pople, 1975). The C8–C11 plane forms a dihedral angle of 84.60 (2)° with the C1–C6 benzene ring. Bond lengths (Allen *et al.*, 1987) and angles are within the normal range and are comparable to those in closely related structures (Fun *et al.*, 2009a,b).

In the crystal packing (Fig. 2), intermolecular N1—H1N1···O4 and C18—H18B···O4 hydrogen bonds (Table 1) link pairs of neighbouring molecules to form chains along the [100] direction; the chains contain *R*_2_^1^(6) ring motifs (Bernstein *et al.*, 1995). Intermolecular C14—H14A···O1 hydrogen bonds further interconnect these chains together to form zigzag layers parallel to the (001). The crystal structure is further stabilized by C—H···π interactions involving the C1–C6 benzene ring (centroid *Cg*1).

### Experimental

Dimethyl 1,4-dihydro-2,6-dimethyl-4-(4-methoxyphenyl)-3,5-pyridine dicarboxylate was prepared according to the Hantzsch pyridine synthesis (Rathore *et al.*, 2009). A mixture of 4-methoxybenzaldehyde (10.0 mmol), methylacetoacetate (20.0 mmol) and ammonium acetate (10.0 mmol) was heated at 353 K for 2 h (monitored by TLC). After the completion of the reaction, the mixture was cooled to room temperature and it was kept for 24 h to get a solid product. The solid formed was washed using diethyl ether. The washed solid was collected separately and the liquid kept for solidification. The purity of the crude product was checked through TLC and recrystallized using acetone and ether.

### Refinement

Atom H1N1 was located in a difference Fourier map and was refined freely [N–H = 0.854 (18) Å]. The remaining H atoms were positioned geometrically [C–H = 0.93–0.98 Å] and were refined using a riding model, with *U*_iso_(H) = 1.2-1.5 *U*_eq_(C). A rotating-group model was applied for the methyl groups. Reflection 010 was partially obscured by the beam stop and hence was omitted. In addition, the most disagreeable reflections 114, 414 amd 248 were also omitted during the refinement.

### Crystal data

**Table:**

C_18_H_21_NO_5_	*Z* = 2
*M**_r_* = 331.36	*F*(000) = 352
Triclinic, *P*1	*D*_x_ = 1.365 Mg m^−^^3^
Hall symbol: -P 1	Mo *K*α radiation, λ = 0.71073 Å
*a* = 7.4106 (3) Å	Cell parameters from 9874 reflections
*b* = 9.5715 (5) Å	θ = 2.8–37.5°
*c* = 11.7771 (6) Å	µ = 0.10 mm^−^^1^
α = 83.029 (1)°	*T* = 100 K
β = 83.834 (1)°	Block, colourless
γ = 77.424 (1)°	0.35 × 0.34 × 0.24 mm
*V* = 806.46 (7) Å^3^	

### Data collection

**Table:**

Bruker SMART APEX DUO CCD area-detector diffractometer	4664 independent reflections
Radiation source: fine-focus sealed tube	4319 reflections with *I* > 2σ(*I*)
graphite	*R*_int_ = 0.019
φ and ω scans	θ_max_ = 30.0°, θ_min_ = 2.7°
Absorption correction: multi-scan (*SADABS*; Bruker, 2009)	*h* = −10→10
*T*_min_ = 0.966, *T*_max_ = 0.976	*k* = −13→13
19600 measured reflections	*l* = −15→16

### Refinement

**Table:**

Refinement on *F*^2^	Primary atom site location: structure-invariant direct methods
Least-squares matrix: full	Secondary atom site location: difference Fourier map
*R*[*F*^2^ > 2σ(*F*^2^)] = 0.037	Hydrogen site location: inferred from neighbouring sites
*wR*(*F*^2^) = 0.110	H atoms treated by a mixture of independent and constrained refinement
*S* = 1.05	*w* = 1/[σ^2^(*F*_o_^2^) + (0.0628*P*)^2^ + 0.2437*P*] where *P* = (*F*_o_^2^ + 2*F*_c_^2^)/3
4664 reflections	(Δ/σ)_max_ = 0.001
226 parameters	Δρ_max_ = 0.48 e Å^−^^3^
0 restraints	Δρ_min_ = −0.22 e Å^−^^3^

### Special details

**Table:**

Experimental. The crystal was placed in the cold stream of an Oxford Cryosystems Cobra open-flow nitrogen cryostat (Cosier & Glazer, 1986) operating at 100.0 (1) K.
Geometry. All esds (except the esd in the dihedral angle between two l.s. planes) are estimated using the full covariance matrix. The cell esds are taken into account individually in the estimation of esds in distances, angles and torsion angles; correlations between esds in cell parameters are only used when they are defined by crystal symmetry. An approximate (isotropic) treatment of cell esds is used for estimating esds involving l.s. planes.
Refinement. Refinement of *F*^2^ against ALL reflections. The weighted *R*-factor wR and goodness of fit *S* are based on *F*^2^, conventional *R*-factors *R* are based on *F*, with *F* set to zero for negative *F*^2^. The threshold expression of *F*^2^ > σ(*F*^2^) is used only for calculating *R*-factors(gt) etc. and is not relevant to the choice of reflections for refinement. *R*-factors based on *F*^2^ are statistically about twice as large as those based on *F*, and *R*- factors based on ALL data will be even larger.

### Fractional atomic coordinates and isotropic or equivalent isotropic displacement parameters (Å^2^)

**Table:**

	*x*	*y*	*z*	*U*_iso_*/*U*_eq_	
O1	0.19666 (9)	0.64176 (7)	0.02070 (6)	0.01872 (14)	
O2	0.45751 (9)	−0.03547 (8)	0.19907 (6)	0.02053 (15)	
O3	0.76827 (9)	−0.08374 (7)	0.16829 (6)	0.01754 (14)	
O4	0.13686 (9)	0.26414 (7)	0.49736 (6)	0.01760 (14)	
O5	0.29405 (9)	0.35204 (8)	0.61485 (6)	0.01934 (14)	
N1	0.78947 (10)	0.17746 (8)	0.42720 (6)	0.01437 (14)	
C1	0.22940 (12)	0.30263 (9)	0.19929 (7)	0.01528 (16)	
H1A	0.1636	0.2291	0.2132	0.018*	
C2	0.17064 (12)	0.41859 (9)	0.11955 (7)	0.01588 (16)	
H2A	0.0667	0.4221	0.0806	0.019*	
C3	0.26760 (11)	0.52967 (9)	0.09799 (7)	0.01404 (16)	
C4	0.42555 (12)	0.52171 (9)	0.15453 (8)	0.01685 (17)	
H4A	0.4930	0.5942	0.1393	0.020*	
C5	0.48191 (12)	0.40434 (9)	0.23414 (8)	0.01621 (17)	
H5A	0.5875	0.3997	0.2718	0.019*	
C6	0.38473 (11)	0.29392 (9)	0.25892 (7)	0.01226 (15)	
C7	0.44663 (11)	0.16962 (9)	0.34963 (7)	0.01211 (15)	
H7A	0.3513	0.1114	0.3631	0.015*	
C8	0.62773 (11)	0.07428 (8)	0.30836 (7)	0.01266 (15)	
C9	0.79129 (11)	0.08547 (9)	0.34450 (7)	0.01310 (15)	
C10	0.63106 (11)	0.23953 (9)	0.49133 (7)	0.01351 (15)	
C11	0.46306 (11)	0.22785 (9)	0.46145 (7)	0.01275 (15)	
C12	0.28140 (14)	0.76387 (10)	0.00644 (9)	0.02215 (19)	
H12A	0.2151	0.8376	−0.0455	0.033*	
H12B	0.4079	0.7363	−0.0243	0.033*	
H12C	0.2782	0.7998	0.0794	0.033*	
C13	0.60706 (12)	−0.01947 (9)	0.22266 (7)	0.01414 (16)	
C14	0.74903 (14)	−0.16612 (11)	0.07708 (9)	0.02279 (19)	
H14A	0.8698	−0.2091	0.0444	0.034*	
H14B	0.6814	−0.1038	0.0188	0.034*	
H14C	0.6831	−0.2403	0.1073	0.034*	
C15	0.28484 (11)	0.28084 (9)	0.52454 (7)	0.01380 (16)	
C16	0.11938 (13)	0.41561 (11)	0.67270 (8)	0.02036 (18)	
H16A	0.1416	0.4659	0.7338	0.031*	
H16B	0.0540	0.3414	0.7037	0.031*	
H16C	0.0462	0.4819	0.6191	0.031*	
C17	0.98251 (11)	0.00466 (9)	0.30817 (8)	0.01614 (16)	
H17A	0.9862	−0.0969	0.3206	0.024*	
H17B	1.0712	0.0281	0.3526	0.024*	
H17C	1.0122	0.0312	0.2282	0.024*	
C18	0.67039 (12)	0.31346 (10)	0.58865 (8)	0.01716 (17)	
H18A	0.5985	0.4101	0.5848	0.026*	
H18B	0.8000	0.3156	0.5830	0.026*	
H18C	0.6379	0.2619	0.6604	0.026*	
H1N1	0.895 (2)	0.1892 (17)	0.4467 (13)	0.030 (4)*	

### Atomic displacement parameters (Å^2^)

**Table:**

	*U*^11^	*U*^22^	*U*^33^	*U*^12^	*U*^13^	*U*^23^
O1	0.0186 (3)	0.0164 (3)	0.0206 (3)	−0.0040 (2)	−0.0063 (2)	0.0048 (2)
O2	0.0147 (3)	0.0233 (3)	0.0262 (3)	−0.0051 (2)	−0.0037 (2)	−0.0088 (3)
O3	0.0143 (3)	0.0177 (3)	0.0218 (3)	−0.0029 (2)	−0.0008 (2)	−0.0080 (2)
O4	0.0111 (3)	0.0237 (3)	0.0187 (3)	−0.0042 (2)	−0.0016 (2)	−0.0036 (2)
O5	0.0127 (3)	0.0272 (3)	0.0193 (3)	−0.0035 (2)	0.0003 (2)	−0.0099 (3)
N1	0.0095 (3)	0.0180 (3)	0.0168 (3)	−0.0037 (2)	−0.0026 (2)	−0.0036 (3)
C1	0.0140 (4)	0.0160 (4)	0.0171 (4)	−0.0055 (3)	−0.0041 (3)	0.0002 (3)
C2	0.0139 (4)	0.0177 (4)	0.0168 (4)	−0.0039 (3)	−0.0055 (3)	0.0001 (3)
C3	0.0140 (3)	0.0141 (3)	0.0131 (3)	−0.0013 (3)	−0.0014 (3)	−0.0005 (3)
C4	0.0166 (4)	0.0161 (4)	0.0192 (4)	−0.0068 (3)	−0.0043 (3)	0.0015 (3)
C5	0.0140 (4)	0.0178 (4)	0.0182 (4)	−0.0056 (3)	−0.0057 (3)	0.0009 (3)
C6	0.0111 (3)	0.0132 (3)	0.0125 (3)	−0.0021 (3)	−0.0014 (3)	−0.0017 (3)
C7	0.0098 (3)	0.0135 (3)	0.0133 (3)	−0.0027 (3)	−0.0021 (3)	−0.0010 (3)
C8	0.0117 (3)	0.0119 (3)	0.0144 (3)	−0.0025 (3)	−0.0016 (3)	−0.0010 (3)
C9	0.0120 (3)	0.0126 (3)	0.0146 (3)	−0.0029 (3)	−0.0010 (3)	−0.0003 (3)
C10	0.0124 (4)	0.0148 (3)	0.0134 (3)	−0.0028 (3)	−0.0021 (3)	−0.0006 (3)
C11	0.0109 (3)	0.0146 (3)	0.0128 (3)	−0.0028 (3)	−0.0012 (3)	−0.0010 (3)
C12	0.0258 (5)	0.0168 (4)	0.0238 (4)	−0.0066 (3)	−0.0043 (3)	0.0043 (3)
C13	0.0141 (4)	0.0123 (3)	0.0161 (4)	−0.0030 (3)	−0.0017 (3)	−0.0006 (3)
C14	0.0205 (4)	0.0232 (4)	0.0270 (5)	−0.0042 (3)	−0.0016 (3)	−0.0131 (4)
C15	0.0131 (3)	0.0148 (3)	0.0132 (3)	−0.0028 (3)	−0.0013 (3)	0.0000 (3)
C16	0.0149 (4)	0.0243 (4)	0.0218 (4)	−0.0022 (3)	0.0021 (3)	−0.0087 (3)
C17	0.0101 (3)	0.0161 (4)	0.0223 (4)	−0.0023 (3)	−0.0014 (3)	−0.0030 (3)
C18	0.0132 (4)	0.0221 (4)	0.0178 (4)	−0.0038 (3)	−0.0037 (3)	−0.0059 (3)

### Geometric parameters (Å, °)

**Table:**

O1—C3	1.3697 (10)	C7—C8	1.5180 (11)
O1—C12	1.4276 (11)	C7—H7A	0.98
O2—C13	1.2163 (10)	C8—C9	1.3565 (11)
O3—C13	1.3516 (10)	C8—C13	1.4698 (11)
O3—C14	1.4433 (11)	C9—C17	1.5040 (11)
O4—C15	1.2226 (10)	C10—C11	1.3603 (11)
O5—C15	1.3461 (10)	C10—C18	1.5026 (11)
O5—C16	1.4416 (11)	C11—C15	1.4637 (11)
N1—C10	1.3843 (10)	C12—H12A	0.96
N1—C9	1.3872 (11)	C12—H12B	0.96
N1—H1N1	0.871 (16)	C12—H12C	0.96
C1—C2	1.3885 (11)	C14—H14A	0.96
C1—C6	1.3938 (11)	C14—H14B	0.96
C1—H1A	0.93	C14—H14C	0.96
C2—C3	1.3925 (12)	C16—H16A	0.96
C2—H2A	0.93	C16—H16B	0.96
C3—C4	1.3912 (12)	C16—H16C	0.96
C4—C5	1.3928 (11)	C17—H17A	0.96
C4—H4A	0.93	C17—H17B	0.96
C5—C6	1.3901 (11)	C17—H17C	0.96
C5—H5A	0.93	C18—H18A	0.96
C6—C7	1.5252 (11)	C18—H18B	0.96
C7—C11	1.5172 (11)	C18—H18C	0.96
			
C3—O1—C12	116.97 (7)	C10—C11—C15	124.91 (8)
C13—O3—C14	115.18 (7)	C10—C11—C7	120.70 (7)
C15—O5—C16	116.31 (7)	C15—C11—C7	114.13 (7)
C10—N1—C9	124.03 (7)	O1—C12—H12A	109.5
C10—N1—H1N1	116.8 (10)	O1—C12—H12B	109.5
C9—N1—H1N1	118.7 (10)	H12A—C12—H12B	109.5
C2—C1—C6	121.53 (8)	O1—C12—H12C	109.5
C2—C1—H1A	119.2	H12A—C12—H12C	109.5
C6—C1—H1A	119.2	H12B—C12—H12C	109.5
C1—C2—C3	119.85 (8)	O2—C13—O3	121.96 (8)
C1—C2—H2A	120.1	O2—C13—C8	123.36 (8)
C3—C2—H2A	120.1	O3—C13—C8	114.65 (7)
O1—C3—C4	124.45 (8)	O3—C14—H14A	109.5
O1—C3—C2	115.92 (7)	O3—C14—H14B	109.5
C4—C3—C2	119.63 (8)	H14A—C14—H14B	109.5
C3—C4—C5	119.49 (8)	O3—C14—H14C	109.5
C3—C4—H4A	120.3	H14A—C14—H14C	109.5
C5—C4—H4A	120.3	H14B—C14—H14C	109.5
C6—C5—C4	121.82 (8)	O4—C15—O5	121.67 (8)
C6—C5—H5A	119.1	O4—C15—C11	123.13 (8)
C4—C5—H5A	119.1	O5—C15—C11	115.20 (7)
C5—C6—C1	117.64 (7)	O5—C16—H16A	109.5
C5—C6—C7	120.39 (7)	O5—C16—H16B	109.5
C1—C6—C7	121.96 (7)	H16A—C16—H16B	109.5
C11—C7—C8	111.44 (6)	O5—C16—H16C	109.5
C11—C7—C6	109.82 (6)	H16A—C16—H16C	109.5
C8—C7—C6	110.88 (6)	H16B—C16—H16C	109.5
C11—C7—H7A	108.2	C9—C17—H17A	109.5
C8—C7—H7A	108.2	C9—C17—H17B	109.5
C6—C7—H7A	108.2	H17A—C17—H17B	109.5
C9—C8—C13	125.21 (7)	C9—C17—H17C	109.5
C9—C8—C7	120.89 (7)	H17A—C17—H17C	109.5
C13—C8—C7	113.77 (7)	H17B—C17—H17C	109.5
C8—C9—N1	118.88 (7)	C10—C18—H18A	109.5
C8—C9—C17	127.61 (8)	C10—C18—H18B	109.5
N1—C9—C17	113.47 (7)	H18A—C18—H18B	109.5
C11—C10—N1	118.71 (7)	C10—C18—H18C	109.5
C11—C10—C18	127.85 (8)	H18A—C18—H18C	109.5
N1—C10—C18	113.43 (7)	H18B—C18—H18C	109.5
			
C6—C1—C2—C3	−0.14 (13)	C10—N1—C9—C8	12.37 (12)
C12—O1—C3—C4	−6.29 (13)	C10—N1—C9—C17	−165.74 (7)
C12—O1—C3—C2	173.39 (8)	C9—N1—C10—C11	−10.00 (12)
C1—C2—C3—O1	−178.08 (8)	C9—N1—C10—C18	169.89 (7)
C1—C2—C3—C4	1.62 (13)	N1—C10—C11—C15	176.50 (7)
O1—C3—C4—C5	178.06 (8)	C18—C10—C11—C15	−3.38 (14)
C2—C3—C4—C5	−1.60 (13)	N1—C10—C11—C7	−9.74 (12)
C3—C4—C5—C6	0.12 (14)	C18—C10—C11—C7	170.39 (8)
C4—C5—C6—C1	1.32 (13)	C8—C7—C11—C10	24.10 (10)
C4—C5—C6—C7	−177.87 (8)	C6—C7—C11—C10	−99.17 (9)
C2—C1—C6—C5	−1.31 (13)	C8—C7—C11—C15	−161.50 (7)
C2—C1—C6—C7	177.87 (8)	C6—C7—C11—C15	75.23 (8)
C5—C6—C7—C11	53.55 (10)	C14—O3—C13—O2	2.95 (12)
C1—C6—C7—C11	−125.60 (8)	C14—O3—C13—C8	−174.88 (7)
C5—C6—C7—C8	−70.05 (10)	C9—C8—C13—O2	173.87 (8)
C1—C6—C7—C8	110.80 (9)	C7—C8—C13—O2	−10.26 (12)
C11—C7—C8—C9	−21.76 (10)	C9—C8—C13—O3	−8.34 (12)
C6—C7—C8—C9	100.90 (9)	C7—C8—C13—O3	167.53 (7)
C11—C7—C8—C13	162.17 (7)	C16—O5—C15—O4	−4.05 (12)
C6—C7—C8—C13	−75.16 (8)	C16—O5—C15—C11	175.05 (7)
C13—C8—C9—N1	−179.23 (7)	C10—C11—C15—O4	−177.44 (8)
C7—C8—C9—N1	5.17 (12)	C7—C11—C15—O4	8.43 (11)
C13—C8—C9—C17	−1.42 (14)	C10—C11—C15—O5	3.48 (12)
C7—C8—C9—C17	−177.01 (8)	C7—C11—C15—O5	−170.65 (7)

### Hydrogen-bond geometry (Å, °)

**Table:**

Cg1 is the centroid of the C1–C6 ring.

**Table:**

*D*—H···*A*	*D*—H	H···*A*	*D*···*A*	*D*—H···*A*
N1—H1N1···O4^i^	0.87 (2)	2.23 (2)	3.0906 (10)	169 (1)
C14—H14A···O1^ii^	0.96	2.54	3.4631 (13)	162
C18—H18B···O4^i^	0.96	2.56	3.4558 (12)	156
C12—H12B···Cg1^iii^	0.96	2.79	3.6549 (11)	151

Symmetry codes: (i) *x*+1, *y*, *z*; (ii) *x*+1, *y*−1, *z*; (iii) −*x*+1, −*y*+1, −*z*.
